# Evidence for confounding eye movements under attempted fixation and active viewing in cognitive neuroscience

**DOI:** 10.1038/s41598-019-54018-z

**Published:** 2019-11-25

**Authors:** Jordy Thielen, Sander E. Bosch, Tessa M. van Leeuwen, Marcel A. J. van Gerven, Rob van Lier

**Affiliations:** Radboud University, Donders Centre for Cognition, Nijmegen, 6525 HR The Netherlands

**Keywords:** Perception, Human behaviour

## Abstract

Eye movements can have serious confounding effects in cognitive neuroscience experiments. Therefore, participants are commonly asked to fixate. Regardless, participants will make so-called fixational eye movements under attempted fixation, which are thought to be necessary to prevent perceptual fading. Neural changes related to these eye movements could potentially explain previously reported neural decoding and neuroimaging results under attempted fixation. In previous work, under attempted fixation and passive viewing, we found no evidence for systematic eye movements. Here, however, we show that participants’ eye movements are systematic under attempted fixation when active viewing is demanded by the task. Since eye movements directly affect early visual cortex activity, commonly used for neural decoding, our findings imply alternative explanations for previously reported results in neural decoding.

## Introduction

Multivariate pattern analysis (MVPA) has become a standard tool for cognitive neuroscience to investigate the representation of cognitive states in the human brain. Compared to its univariate counterpart, MVPA uses distributed patterns of activity to predict regressors of interest (‘decoding’). As a result, it is more sensitive to weak within-subject trial-by-trial variance^1^. From functional magnetic resonance imaging (fMRI) responses, passively perceived and actively attended stimulus orientation can be decoded^2^, as well as unconsciously perceived stimulus orientations^3^ and those actively held in visual working memory^4^. Similarly, passively perceived and actively attended motion direction can be decoded from fMRI responses^5^, as well as actively imagined motion direction^6^. Finally, passively perceived objects can be decoded from fMRI responses^7–9^, but also objects^10^ and scenes^11^ that are actively held in working memory. From electrophysiological measures like magnetoencephalography (MEG) and electroencephalography (EEG) perceived orientation^12^ and perceived motion direction have also been classified^13^.

In all of these studies, and as common practice in cognitive neuroscience in general, participants are asked to fixate on a fixation dot during the experiments. This is considered necessary to reduce any potential confounding role of eye movements. Eye movements cause changes at the neuronal level, since they have a direct effect on early visual cortex due to its retinotopic organization and typically small receptive field sizes. Therefore, a shift in the image caused by an eye movement will be reflected as retinal slip, and subsequently as a shift in the location of activity at the neuronal level. Evidence supporting this direct link between eye movements and neural activity changes comes from a study that showed similar neural changes to artificial image shifts, small voluntary eye movements, and microsaccades^14^. Additionally, low-level visual areas, frequently used in decoding studies, were sensitive to eye movements, while high-level visual areas remained eye movement invariant^15^. On the contrary, in the absence of visual stimuli, stimulus-specific gaze patterns were shown to reactivate representations in higher-level areas^16^. Apart from these effects, eye movements may also affect brain activity indirectly, as motor plans are prepared for saccades.

Importantly, participants make eye movements even under attempted fixation. These so-called fixational eye movements are small jerk-like movements around the point of fixation and comprise of microsaccades, tremor, and drift^17^. The presumed role of fixational eye movements is to prevent perceptual fading - under perfect fixation retinal input will remain perfectly stable, causing neural adaptation and hence a fading percept. Hence, fixation during neuroimaging experiments may not exclude confounding effects of systematic eye movements on neural activity.

Such fixational eye movements contaminate neural data in two ways. Firstly, when the eye movements are random by nature, they add additional noise to the data reducing the signal to noise ratio and increasing the false negative rate (type II error). In this case, the right choice of fixation target can improve fixation stability^18^. Secondly, when the eye movements are systematic (i.e., they covary with the experimental conditions), they cause false positives (type I error). This poses a more serious problem, as one might interpret an effect as brain-related, while it was actually caused by artefacts induced by eye movements, or at least eye movements contributed partially to the effect. It is this second type of eye movements that we investigate in the current study.

Confounding fixational eye movements have already been reported in several MEG decoding studies on visual working memory^19,20^ and on visual imagery^21^. A common aspect of those studies was that participants were required to actively attend to the stimuli. This active involvement might have induced systematic eye movements, but this relation to the type of task was not directly tested in those studies.

Indeed, effects of attention have been reported on both involuntary fixational eye movements and voluntary goal-oriented eye movements. For instance, the direction of spatial attention^22–24^ as well as the direction of perceived apparent motion^25^ could be predicted from the direction of microsaccades. Additionally, stronger feature-based^26,27^ and spatial^24^ attention decreased the microsaccade rate, but at the same time the microsaccades became more informative about the locus of attention. It should be noted here that biases in such small amplitude eye movements did not only arise in a stimulus-driven fashion, but also arose when the stimulus was not physically presented but was held in visual working memory^28^. Furthermore, gaze patterns for the same stimulus varied substantially under varying cognitive goals^29^. In line with this finding, eye movements were shown to be directed to the salient parts of the visual scene^30,31^. In sum, these studies show that active task involvement could cause systematic eye movements.

In our previous work, we investigated the potential confounding role of eye movements in a passive task, but we found no evidence for any systematic eye movements under passive viewing^32^. The passive fixation task means that participants could have potentially ignored the stimuli. In the current study, we investigate whether active versus passive viewing of stimuli could be an explanation for the occurrence of systematic eye movements. Since many paradigms as used throughout neuroimaging studies rely on active paradigms, this study forms an important follow-up on our previous work, of significance to investigate the potential confounding role under an active task.

## Results

We conducted an experiment using a within-subject design to investigate within-subject effects of a subtle task manipulation and to exclude the potential role of differences between individual participants. We investigated the role of eye movements in orientation decoding, as this is a pioneering framework in neural decoding and is still used for vision sciences in fMRI, EEG, and MEG research. Importantly, task effects on orientation decoding have not yet been demonstrated.

In the pioneering study on orientation decoding, participants passively perceived eight differently oriented square-wave gratings while they fixated on a fixation dot. The study showed that passively perceived stimulus orientation can be decoded from early visual cortex as measured by fMRI^2^.

**Figure 1:**
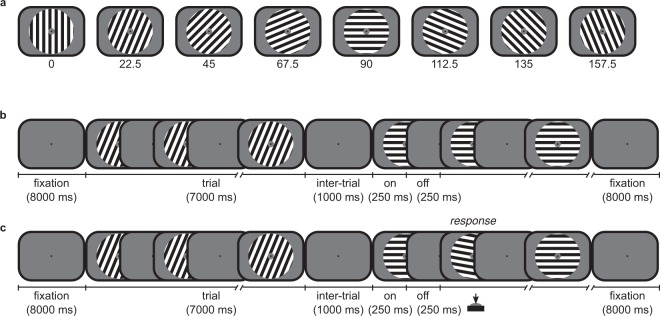
Eye movements were recorded while participants passively or actively perceived differently oriented square-wave gratings while fixating at a fixation dot. (**a**) The experiment presented eight differently oriented (0 to 180 degrees in steps of 22.5 degrees) square-wave (spatial frequency of 1.5 cycles per visual degree, 100% contrast) gratings (outer radius of 10.0 and inner radius of 1.5 visual degrees) presented on a mean-luminance grey background. Throughout the entire experiment, participants were instructed to maintain fixation at a fixation dot (outer radius of 0.1 visual degrees). (**b**) Each of twelve runs of the passive session started with 8 seconds of fixation and finished with 8 seconds of fixation. In between, sixteen 7-second trials were presented with 1 second inter-trial interval. A trial presented one oriented grating at 2 Hertz (i.e., 250 ms ‘on’, 250 ms ‘off’), where each ‘on’ period presented the grating with a uniform random phase. (**c**) The active session was similar to the passive session, but on 12.5 percent of trials one ‘on’ period within a trial presented the oriented grating with a 10 degree perturbation (clockwise or counter-clockwise). Participants were instructed to detect these perturbed trials upon which they had to press a button.

In the current study, participants also perceived eight differently oriented square-wave gratings (Fig. 1a). In contrast to the previous studies^2,32^, participants performed both a passive session (Fig. 1b) as well as an active session (Fig. 1c) on separate days in counterbalanced order. In both sessions, participants were instructed to fixate on a fixation dot while their fixational eye movements were recorded. The passive session is a replication of the pioneering work on orientation decoding^2^ and our previous work^32^. The active session was similar to the passive one, except for a subtle task manipulation that forced participants to actively attend to the presented stimulus in the active session, while participants passively viewed the stimuli in the passive session and could thus ignore them. Specifically, in the active condition, the orientation of the presented stimulus was slightly perturbed on 12.5 percent of the trials, upon which participants had to make a button press.

**Figure 2:**
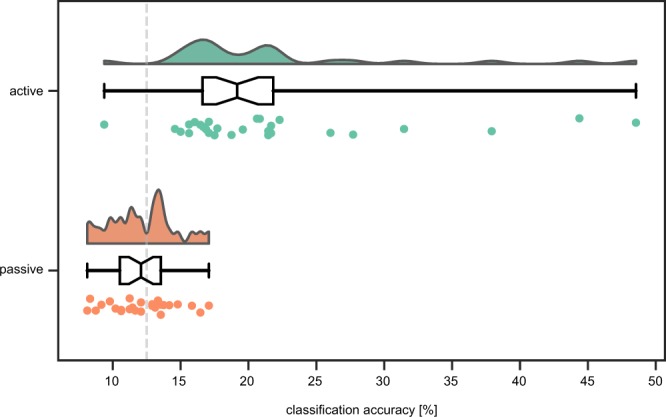
Eye movements are systematic in the active but not the passive session. A linear support vector machine was trained to classify stimulus orientation from eye movements. The classification accuracies (i.e., percentage of correctly classified trials) are shown as distributions (top), box plot (middle), and individual data points (bottom) for both active (green) as well as passive (orange) session. The classification accuracies were significantly larger in the active (median = 19.2%) than in the passive (median = 12.1%) session (*t* = 4.0, *n* = 28, *p* < 0.01, Wilcoxon signed-rank test for two related paired samples). The gray dashed line illustrates chance level (12.5 percent). For detailed results see main text.

We analyzed the recorded eye-tracking data and attempted to decode the orientation of the presented stimulus given the eye movement time series (Fig. 2). The classification accuracies were significantly larger in the active (median = 19.2%) than in the passive (median = 12.1%) session (*t* = 4.0, *n* = 28, *p* < 0.01, Wilcoxon signed-rank test for two related paired samples). Moreover, the classification accuracies in the passive session (minimum 8.1%, maximum 17.1%) were not significantly different from chance level (*t* = 164.0, *n* = 28, *p* = 0.37, Wilcoxon singed-rank test), while the decoding in the active session (minimum 9.4%, maximum 48.5%) was significantly different from chance level (*t* = 4.0, *n* = 28, *p* < 0.01, Wilcoxon signed-rank test). At the level of the individual participants the differences between the active and passive session were equally clear: in the passive session none of the participants’ eye movements allowed orientation decoding accuracy higher than chance level (*p* > 0.05, permutation test with 1000 permutations), while in the active session this was achieved for sixteen out of twenty-eight participants (*p* < 0.05, permutation test with 1000 permutations). It should be noted here that decoding of the active session was performed without the task trials (i.e., the 12.5 percent of trials containing a perturbed orientation), as these could contain eye movements caused by the orientation perturbation. Finally, there was no effect of session order (*t* = 43.0, *n* = 14, *p* = 0.55, Wilcoxon signed-rank test), so the difference in decoding accuracy between the active and passive session was not different between the participants performing the active session first and subsequently the passive session, and those who did the sessions in reversed order.

Considering that the task manipulation drives the difference between the active and passive session, one might expect that those participants who more actively monitor the stimulus will show more systematic eye movements, which in turn will increase the classification accuracy. In general, participants performed the task well in the active condition (average 93.5%, minimum 83.9%, maximum 98.4%). To test the effect of task performance on systematic eye movements, we computed the Spearman correlation coefficient between the task accuracies and classification accuracies. There was no significant correlation between the task performance and decoding performance (*ρ*(26) = 0.08, *p* = 0.70, 95% CI [−0.32, 0.45]). Since the task trials were infrequent, we also correlated the sensitivity (*ρ*(26) = 0.13, *p* = 0.51, 95% CI [−0.27, 0.49])) and specificity (*ρ*(26) = −0.26, *p* = 0.57, 95% CI [−0.48, 0.29]), but both resulted in non-significant correlations.

As an additional analysis, we inspected the average eye movement amplitude. First, the eye movement amplitude in the passive session (median 0.2, minimum 0.1, maximum 0.9 visual degrees) was significantly different (*t* = 11.0, *n* = 28, *p* < 0.01, Wilcoxon signed-rank test) than the amplitude in the active session (median 0.3, minimum 0.1, maximum 2.9 visual degrees). The eye movement amplitude did not correlate with task accuracy (*ρ*(26) = −0.13, *p* = 0.51, 95% CI [−0.49, 0.27]), sensitivity (*ρ*(26) = −0.17, *p* = 0.38, 95% CI [−0.52, 0.23]), or specificity (*ρ*(26) = 0.13, *p* = 0.52, 95% CI [−0.27, 0.49]). However, eye movement amplitude did significantly correlate with classification accuracy (*ρ*(26) = 0.51, *p* = 0.01, 95% CI [0.15, 0.75]), so that larger eye movements resulted in higher classification accuracies.

**Figure 3:**
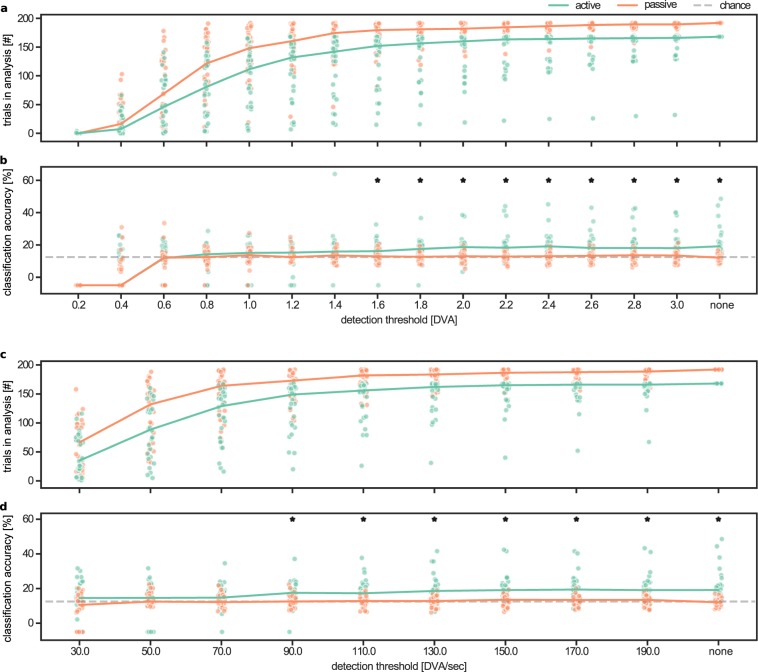
Removing trials with large saccades reduces the confounding effect of eye movements. Two ways of detecting deviating eye movements are shown. In the first method (**a**,**b**) those trials are removed in which the eye was a certain degrees of visual angle (DVA) away from the fixation point. In the second method (**c**,**d**) those trials are removed in which at least one saccade is made. Saccades are detected by thresholding the eye movement velocity. By increasing the thresholds, more trials stay in the analysis that might contain systematic eye movements (**a**,**c**), which in turn increases classification accuracy in the active condition (**b**,**d**). An asterisk denotes a significant difference between the active session and the passive session (*P* < 0.05, Bonferroni corrected). Solid lines show the session-specific median values and dots represent individual datapoints. The gray dashed line represents chance level decoding (i.e., 12.5 percent). Note, a classification accuracy of −5% denotes that too many trials were removed to be able to perform decoding. Additionally, the untreshholded results are shown at the end marked as ‘none’ in all figures, which are the results from the original analysis as in Fig. 2.

In line with the positive correlation of eye movement amplitude with classification accuracy, we conducted a decoding analysis while removing trials that were contaminated with saccades. The detection of saccades was performed in two ways. In the first analysis, we removed those trials in which the eye position was further away from fixation than a certain threshold (i.e., a circle around fixation beyond which trials would be removed). Note that, with this approach participants could still make saccades within the boundaries. In the second analysis, we removed those trials in which the eye movement velocity exceeded a certain threshold (i.e., a saccade was made). The results are shown in Fig. 3 and clearly show that for certain thresholds it is possible to remove trials in which participants did not fixate well, which in turn makes the decoding effect disappear in the active session. Note that for some participants most if not all trials had to be removed for certain thresholds, which makes decoding impossible at all because of the few remaining datapoints.

## Discussion

Thus, stimulus-orientation could not be decoded from eye movements in the passive session, but importantly, they could be decoded from eye movements in the active session for the same participants. This implies that stimulus-dependent eye movements arise when participants are actively involved in monitoring the stimuli as compared to a passive session. The absence of orientation decoding in the passive session is in line with our previous study^32^. Thus, under active viewing but not under passive viewing, orientation decoding is contaminated by eye movements.

We suggest several hypotheses as to why such stimulus-dependent eye movements might emerge. First, the active task might be best solvable at certain locations of the stimulus. For oriented gratings the difference in orientations is the largest at the outer edges of the stimulus along the grating direction. Therefore, participants might have made goal-oriented voluntary saccades along the stimulus orientation to optimize task performance even though they were instructed to fixate throughout the experiment. Second, participants might make subtle eye movements like microsaccades in the direction that changes the retinal input the most to optimize prevention of neuronal adaptation. In the case of gratings, this is orthogonal to the orientation of the grating. It should be noted however, that such microsaccades would happen in both passive as well as active session. Still, the magnitude of these might interact with session type. Third, the spatial phase of the oriented grating is randomized over individual presentations during a trial. This is done to prevent individual pixels of being descriptive for orientations, but may introduce an illusory percept of the grating moving over the screen orthogonal to its orientation. This percept of motion might in turn cause systematic eye movements orthogonal to the orientation of the grating, since the direction of perceived apparent motion is related to the direction of microsaccades^25^. Also here, one should note that these type of eye movements would emerge in both sessions, but might again interact with session type. Finally, so far we have discussed eye movements as the cause for brain activity, but it might as well be the other way around. Specifically, neural activity might be elicited by the low-level stimulus properties – like orientation that can be decoded – and might in turn be the cause of stimulus-dependent eye movements rather than its consequence.

**Figure 4:**
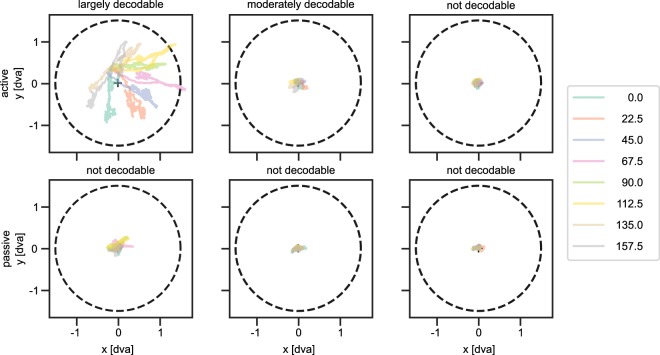
Eye movements show clear systematic patterns only for the active condition in some participants. (**a**) The top row shows the eye position over the course of a trial in the active session. (**b**) The bottom row shows the eye position over the course of a trial in the passive session. Colours represent trials belonging to one of eight orientations, and are those as shown in Fig. 1a. The solid lines represent averages over repeated presentations of the same orientation. The dashed line represents the inner annulus of the square-wave gratings, which had a radius of 1.5 visual degrees. Additionally, averages are computed for those participants with higher than 30% classification accuracy in the active session (N = 4) shown in the first column, those with lower than 30% classification accuracy but still significant decoding in the active session (N = 12) in the middle column, and those that were not decodable in the active session (N = 12) in the right column. Note that the passive session was never decodable, but for comparison the same groups are shown. This figure illustrates that for some participants there is a clear pattern in their eye movements, while for others this pattern is less clear. Additionally, these patterns remain completely absent in the passive sessions, even for those showing large patterns in the active session.

We inspected the eye movement patterns and found that those participants that showed largely decodable eye movements (i.e., a classification accuracy higher than 30%) showed large eye movements along the direction of the presented stimulus (Fig. 4). Those with moderately decodable eye movements showed smaller biases, but still in the direction of the orientation. This might suggest that participants adopted an optimal strategy to solve the task in the active session, even though they were told to fixate. However, as mentioned before, there was a correlation between classification accuracy and eye movement amplitude, but no correlation between classification accuracy and task accuracy. This observation makes it less likely that the strategy of solving the task had an influence on the systematicity of the eye movements. Hence, future research is needed to further investigate how and why these eye movements emerge.

Given the large eye movements as shown in Fig. 4 and the fact that it was possible to reduce the confounding effect by removing trials that contain saccades, we believe large (maybe voluntary) eye movements under attempted fixation drive the reported effect. Still, we cannot exclude the possibility that microsaccades also contribute to the classification accuracy. We detected microsaccades using the methodology as described by Engbert^33^, but found no evidence for direction or amplitude of microsaccades to covary with stimulus orientation.

Our results show that only a subset of participants showed eye movements that covary with stimulus orientation. This raises an important question about what could explain the variability in classification accuracy across participants. Again, task performance did not explain these differences. Still, the strategy used to solve the task and the (in)ability to fixate sufficiently, as well as other individual differences might explain the differences. Unfortunately, the present experiments preclude further investigation of these factors. Additionally, although we have shown the emergence of confounding eye movements using only a subtle task manipulation, future research is needed to test other stimulus types (e.g., kinematograms or natural scenes), stimulus characteristics (e.g., duration or contrast), or paradigms (e.g., a working memory paradigm).

We propose several ways to deal with confounding effects of eye movements in neuroimaging studies. We showed that the ability to decode stimulus orientation decreased when removing trials with fixation away from the fixation point or removing trials that contained saccades. First, one could detect such trials in real-time as a measure of how well participants fixate and have them redo a trial when fixation was not stable enough. Second, one could simply remove these trials from post-hoc analysis, but this might result in losing large parts of the data. Do note that for these two options to work, a suitable detection threshold needs to be found, which might be subject and task dependent. Third, one could use the eye movement patterns as nuisance regressors in the analysis. However, one should be careful still, as such regression would only remove the linear effects of eye movements, and would ignore other processes involved in for instance saccade planning. Finally, in reporting results, one could measure the mutual information in the brain activity over eye movements, as was carried out by Quax and colleagues^20^.

In conclusion, based on the current study, we advise researchers to monitor eye movements always by recording and extensively analyzing eye tracking data alongside the brain activity. Within EEG and MEG studies, it is shown that recording the electrooculogram (EOG) is not sufficient to deal with confounding eye movements^20^. Fixational eye movements might be easily missed and should be analyzed extensively (post-hoc) to investigate the potential confounding effects of eye movements. In general, studies in neural decoding should be aware of the potential confounding role of eye movements under active viewing conditions. Our results suggest that these eye movements are uncontrollable in the sense that they play a functional role in task execution. To what extent specific stimulus paradigms and tasks used throughout cognitive neuroscience are affected and to what degree this affects previously drawn conclusions remains to be analyzed in future research.

## Methods

### Participants

Twenty-nine university students (aged 19–31; 22 females) from the Radboud University participated in the experiment. Inclusion criteria were age (18–31), handedness (right), and vision capabilities (uncorrected, normal). Exclusion criteria were any history with epilepsy or claustrophobia. All participants gave written informed consent prior to the experiment and received payment or course credit after the experiment. The experimental procedure and methods were approved by and performed in accordance with the guidelines of the local ethical committee of the Faculty of Social Sciences of the Radboud University. Participant 15 did not complete the full experimental design and was therefore left out of the analysis.

### Materials

The stimuli were full contrast (100 percent) black-white square-wave gratings of eight possible orientations ranging from 0 to 180 degrees in steps of 22.5 degrees. The spatial frequency was 1.5 cycles per degree of visual angle and the spatial phase was randomized every presentation. The stimuli had an outer radius of 10.0 degrees and an inner radius of 1.5 degrees of visual angle and were presented at the center of a mean-luminance grey background. A fixation dot was presented at the center of the screen with an outer radius of 0.1 degree of visual angle and an inner radius of 0.075 degree of visual angle.

The experiment was run on a Windows 10 desktop PC running Python version 2.7.14 and the PsychoPy library, version 1.90.2. The stimuli were presented on a 24-inch BenQ XL2420Z monitor with a 60 Hertz frame rate and 1920 × 1080 pixel resolution. The monitor subtended 39.1 degrees of visual angle horizontally and 22.0 degrees of visual angle vertically. An EyeLink 1000 Plus (SR Research, Ltd.) desktop-mounted eye tracker was used to record binocular eye positions and pupil dilation with the 35-millimeter lens at a sample rate of 1000 Hertz. The eye tracker was positioned just below and in front of the monitor at a distance of 55 centimeters from the participant’s eyes. The participant’s head position and viewing distance were fixed at 65 centimeters from the monitor with a chin and forehead rest.

Preprocessing and data analyses were performed using Python 3.7.1. Custom analysis pipelines were made using the NumPy library (version 1.15.4) and SciPy library (version 1.1.0) for scientific computing, and the Scikit-Learn library (version 0.20.1) for machine learning.

### Procedure

The experimental paradigm was similar to that of Kamitani and Tong^2^ and Thielen and colleagues^32^, though some adaptations were made to incorporate the active session. The experiment contained two sessions, which were run on separate days. Participants with an odd participant number completed the active session in the first session and the passive session in the second session, while participants with an even participant number completed the two sessions in reverse order.

Both sessions contained twelve runs, each presenting all eight orientations twice for a total of sixteen trials per run. A run was initiated by a button press and started as well as ended with 8 seconds of fixation during which only the fixation dot was presented at the center of the screen. In between these fixation periods, the sixteen trials were presented sequentially in random order with an inter-trial interval of 1 second. Throughout the inter-trial interval, only the fixation dot was presented. In each trial, one of eight stimuli was presented at 2 Hertz (on/off for 250 milliseconds) for 7 seconds. During the’on’ period the stimulus was presented with a random phase together with the fixation dot, while during the’off’ period only the fixation dot was presented.

The active session differed slightly from the passive session. In the active session, 12.5 percent of the trials were used as task trials. In a task trial the orientation of the stimulus was perturbed for one ‘on’ period (250 milliseconds) in between 1 to 6 seconds of the trial. The perturbation was always 10 degrees and could be clock-wise or counter clock-wise with equal probability. Task trials occurred pseudo-randomly so that there were three task trials for each orientation in the entire session, randomly allocated to runs. In this way, both the timing during a trial, as well as the number of task trials within runs seemed random to the participant. Participants had to push a button with their right index finger upon detection of a deviant stimulus.

Prior to a session, participants were told to fixate throughout runs regardless of the session type. Participants who performed the passive session first, were prior to the active session told that now there was a second task on top of fixation, namely detecting the deviant stimuli. Participants who performed the active session first, were told prior to the first session that there are two tasks being fixation and detection of the deviants, and were told prior to the passive session that the deviant stimuli will not happen anymore so that only the fixation task was left. In both sessions, participants were shown an example run, which included a task trial in the active session, so that they knew what to expect.

### Preprocessing

The binocular eye positions and pupil dilation recordings were acquired at a sampling rate of 1000 Hertz and saved for offline analysis. Missing values caused by eye blinks were replaced by median values (i.e., the fixation point). Subsequently, the data were low-pass filtered using a Butterworth filter of order 5 and a cutoff frequency at 100 Hertz. The data were then down sampled to 256 Hertz. Finally, the data were sliced to individual trials of 7 seconds locked to the onset of the first stimulus presentation in a trial. Medians were subtracted from individual runs to center the data around the fixation point and remove any biases within runs.

### Analysis

We carried out a decoding analysis with the attempt to identify the presented stimulus from the eye movement data from the active and passive session separately. For this, we computed the averages and standard deviations of the recorded binocular Cartesian eye positions and pupil dilation, yielding 12 features for each trial. We removed the task trials in the active session for a total of 168 remaining trials in the active session (i.e., 21 trials per orientation) and 192 trials in the passive session (i.e., 24 trials per orientation).

We used 10-fold stratified cross-validation to estimate a generalization performance. For each fold, training and validation data were normalized by removing the median and by scaling according to the inter-quartile range fitted on the training data. Subsequently, a linear support vector machine (SVM) with regularization parameter *C* = 0.1 was trained on the training data and applied to the validation data to estimate the classification accuracy. We computed the generalization performance as the average classification accuracy over folds.

Statistical significance within participants was estimated using a permutation test. For this, the distribution under the null was estimated by running the decoding pipeline 1000 times with permuted labels. Classification was considered significantly above chance level if it was above 95% of the null distribution.

Statistical significance between the two sessions (active versus passive) was estimated using the Wilcoxon signed-rank test for two related paired samples. Statistical significance within a session (active or passive) was assessed using the Wilcoxon signed-rank test with difference scores with chance level (12.5%). We used an alpha threshold of 5% to consider statistical significance.

## Data Availability

All raw data and analysis scripts are made publicly available at the Donders Data Repository (http://hdl.handle.net/11633/aacubhf3).
